# Facile thermal exfoliation of Cu sheets towards the CuO/Cu_2_O heterojunction: a cost-effective photocatalyst with visible-light response for promising sustainable applications†

**DOI:** 10.1039/c9ra06837f

**Published:** 2019-10-17

**Authors:** Yixuan Li, Xi Chen, Li Li

**Affiliations:** College of Chemistry and Chemical Engineering, Qiqihar University Qiqihar Heilongjiang 161006 P. R. China qqhrll@163.com qqhrlili@126.com; College of Chemistry and Chemical Engineering, Harbin Institute of Technology Harbin Heilongjiang 150090 P. R. China; College of Materials Science and Engineering, Qiqihar University Qiqihar Heilongjiang 161006 P. R. China

## Abstract

Unlike semiconductors, metals, especially the Cu element, usually show preeminent ductility and thermal expansivity; accordingly, herein, we report thermal treatment of a commercial copper sheet in air to obtain a black mass, *i.e.* CuO/Cu_2_O with a heterojunction, which has been proven to be a photocatalyst. Rapid cooling of the hot sheet detached/knocked off the oxidized layers from the sheet as a powder. The extent of the formation of copper oxides was determined by electrochemical impedance spectroscopy and transient photocurrent responses; in addition, other physical methods for the characterization of the catalyst were performed, and the catalyst exhibited applications in the mineralization of the rhodamine B (RhB) dye, a potential pollutant in water bodies. Moreover, the well-matched band structures of CuO/Cu_2_O manifested that CuO and Cu_2_O could serve as reduction sites and hole oxygen sites to generate the ·O_2_^−^ and ·OH species, respectively, which were subsequently affirmed by the DMPO electron spin resonance technique. In this study, we proposed a novel perspective towards the design of heterogeneous photocatalysts for organic pollutant treatment through a convenient solid-phase synthesis rather than a complicated liquid-phase synthesis. In addition to this, the resultant CuO/Cu_2_O photocatalyst proposed herein might inspire the researchers to focus on the preparation of more cost-effective photocatalysts for commercialization rather than complicated nanocomposites for pure theoretical research.

## Abstract 2

A CuO/Cu_2_O heterojunction with broad visible light response and enhanced photogenerated carriers properties was synthesized *via* a facile one-step thermal exfoliation process from a commercial copper sheet.

## Graphical abstract

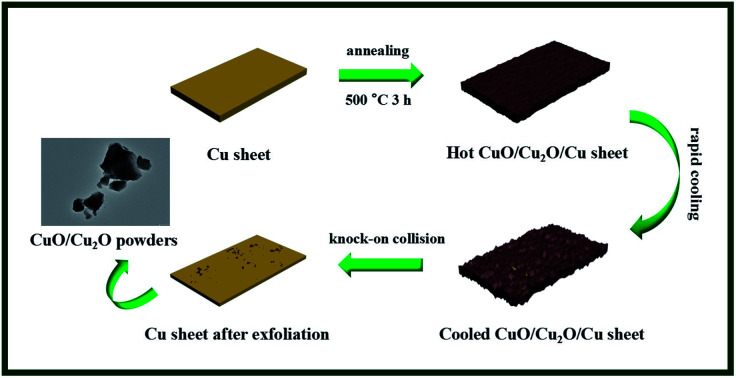

## Introduction

Among the affluent abatement strategies reported to date, semiconductor photocatalysis has attracted significant attention.^1–3^ As the commercially available photocatalyst representative, TiO_2_ has been widely applied; however, some inherent defects, such as ultraviolet light absorption, low quantum yield, high cost, and complex preparation process, enormously limit its large-scale applications.^4,5^ As a result, in recent years, a series of novel heterogeneous photocatalysts, such as AgCl/Ag_3_PO_4_/g-C_3_N_4_,^6^ Pd-Ag/AgBr/TiO_2_,^7^ AgI/Bi_2_Sn_2_O_7_,^8^ and Ag-CQDs/g-C_3_N_4_,^9^ with broad visible-light harvesting ability have been exploited, which show outstanding photocatalytic performances; however, we believe that some advanced photocatalysts may not meet the requirements of industrialization for the following reasons. On the one hand, they ordinarily contain noble metals (Ag, Au, and Pd), which dramatically increase the manufacturing cost. On the other hand, the constructed heterojunctions are fabricated by a complex synthesis method involving multiple steps in most cases.^10–12^ Hence, it should be an interesting challenge to pursue a more low-cost and convenient strategy for the synthesis of composite photocatalysts with a visible-light response.

Copper-oxide compounds, with respect to commercial production, appear to be more advantageous when compared with conventional photocatalysts owing to their highlighted superiorities such as high abundance, simple preparation, low cost, and element nontoxicity.^13–15^ Especially, they usually feature small band gaps for visible-light absorption without the decoration of any photo-sensitizers.^16,17^ Nowadays, large numbers of researchers are aiming to develop Cu-based composites and have harvested many achievements in this regard.^18–20^ Although these studies are very innovative, there is still a lot of room for improvement. Actually, the synthesis processes of most heterogeneous photocatalysts belong to the liquid-phase reaction.^21–23^ For example, Li *et al.* reported that the CuO/Cu_2_O heterostructure could be prepared *via* a facile liquid-phase process using PVP as a surfactant, and this heterostructure showed a favorable photocatalytic performance for dye degradation.^24^ Thus, the introduced PVP could be attached on the surface of the photocatalyst such that to further control the morphology of the photocatalyst. However, the surfactant also hampered the contact between the photocatalyst and the dye, hindering the achievement of desired photocatalytic activity.

Therefore, to accommodate the requirements of large-scale application, simplifying the synthetic procedure and aggrandizing the photocatalyst yield may be of great significance, especially for solid-phase synthesis. However, to the best of our knowledge, limited studies have been conducted in this regard. Interestingly, it is worth mentioning that Li *et al.* have discovered that the Cu/Cu_2_O/CuO heterojunction can be prepared by a one-step calcination process in air using the Cu net as the matrix, and this heterojunction successfully degrades organic pollutants under visible light irradiation.^25,26^ However, there are still some drawbacks. For instance, the generated photocatalyst is in the form of a net rather than powder; this means low-effective catalytic quality and difficulty in the separation and storage of the photocatalyst. Moreover, both Cu and CuO in the composite can capture photogenerated electrons and thus hinder the effective oriented transfer of carriers during photocatalysis. Therefore, the exploitation of a new strategy for the convenient preparation of copper-oxide composites with optimized carrier behaviors and broad visible-light responses can be a worthwhile challenge to conquer.

**Scheme 1:**
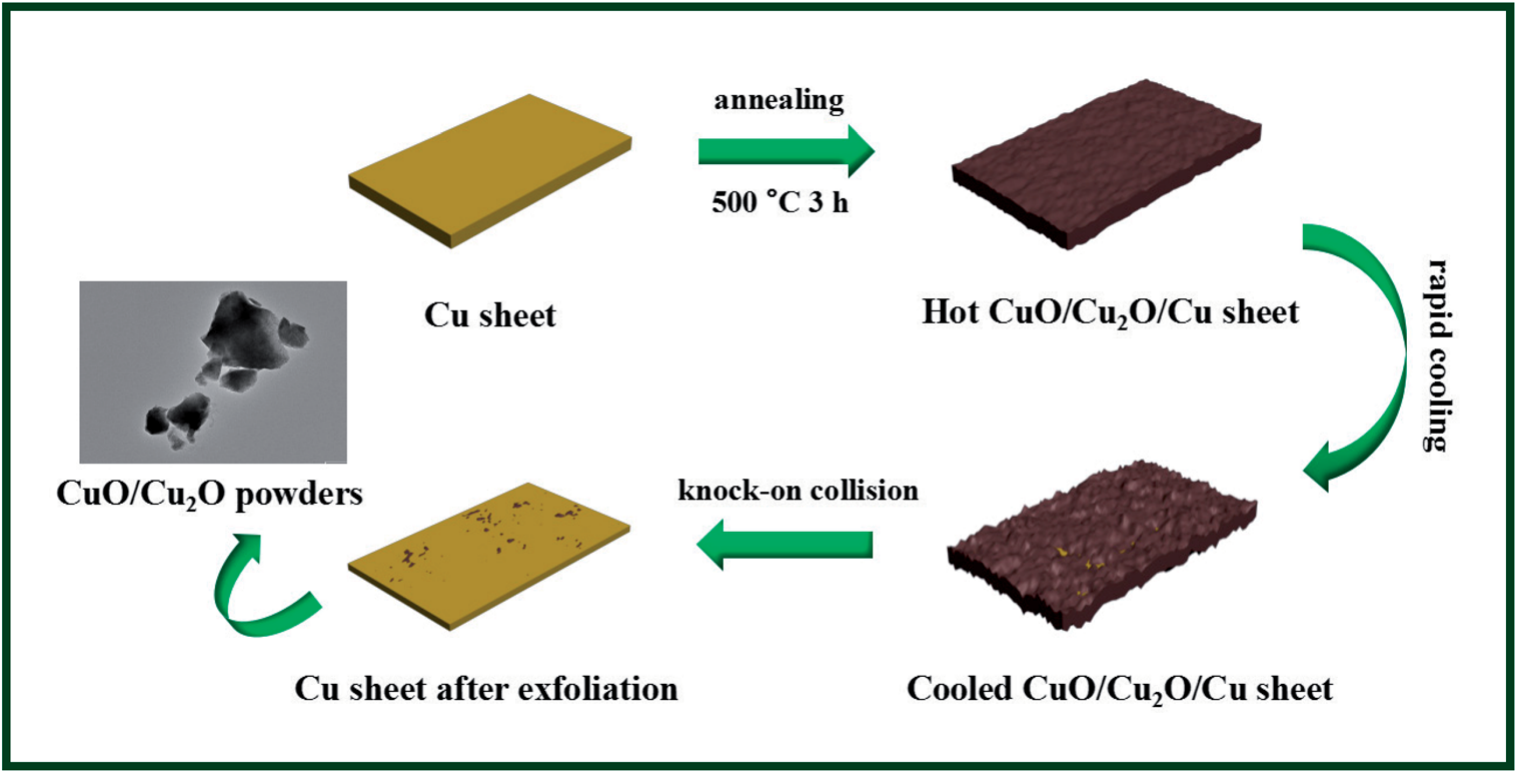
The CuO/Cu_2_O composites were synthesized *via* a facile one-step thermal exfoliation process from a commercial copper sheet.

Herein, a facile strategy was developed for the one-step thermal exfoliation of a commercial Cu sheet to fabricate the CuO/Cu_2_O heterojunction, which absolutely differed from the conventional liquid-phase reaction. Moreover, the derived CuO/Cu_2_O composites could be easily separated from the Cu sheet *via* a knock-on collision process utilizing the huge differences between the thermal expansivity and ductility of the metal and semiconductor, and the morphological evolution is illustrated in Scheme 1. Specifically, we first heated the Cu sheet at 500 °C, which would cause the sheet to be dilated and then oxidized by the hot air flow, and consequently, the CuO/Cu_2_O composites were generated on its surface. After annealing, the resulting CuO/Cu_2_O/Cu sheet was rapidly cooled down by the air flow at room temperature. At this moment, the inside copper sheet with the preeminent thermal expansivity would obviously shrink, whereas the outside CuO/Cu_2_O would not. This would leave a certain interval between them, and the following interface separation would be achieved. The CuO/Cu_2_O composites with poor ductility began to spontaneously splinter without the compact support of the Cu sheet. Subsequently, the CuO/Cu_2_O/Cu sheet was repeatedly knocked on, and then, the CuO/Cu_2_O photocatalyst powders were obtained. Ultimately, the photocatalytic performances of CuO/Cu_2_O were investigated towards organic pollutant removal (RhB) under visible light irradiation, and these composites showed favorable stability and reusability for promising large-scale applications.

## Results and discussion

### Characterization of the CuO/Cu_2_O nanocomposites

**Fig. 1:**
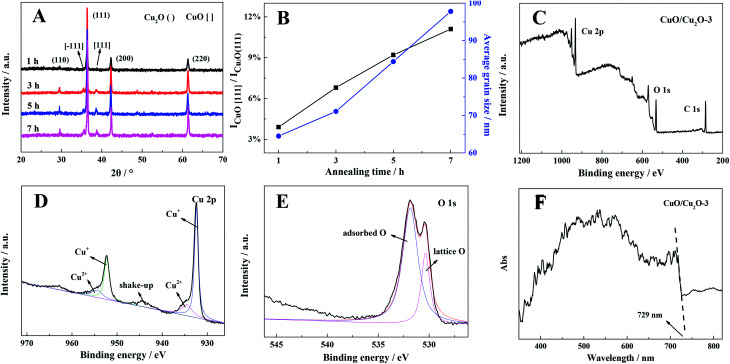
(A) XRD patterns of different samples; (B) the variations in ICuO[110]/ICu_2_O(111) and average grain sizes influenced by annealing time over different samples; (C) XPS spectra of the CuO/Cu_2_O-3 composite, (D) Cu2p, and (E) O1s; and (F) UV-Vis DRS spectrum of the CuO/Cu_2_O-3 composite.

At first, X-ray diffraction (XRD) was carried out to explore the phase structure of the as-prepared samples, and the results are shown in Fig. 1A. The diffraction peaks at the 2*θ* values of 29.6°, 36.4°, 42.2° and 61.3° corresponded to the (110), (111), (200) and (220) facets, indicating that the crystal structures of the samples well indexed to that of cubic Cu_2_O (JCPD standard card no. 65-3288).^25^ The diffraction peaks at 35.6° and 38.7° are ascribed to the (−111) and (111) diffraction phases of monoclinic CuO (JCPD standard card no.48-1548), respectively.^27^ No diffraction peaks of other phases could be observed; this confirmed high purity of the products. Moreover, as illustrated in Fig. 1B, the relative content of CuO increased with the increasing annealing time, which dramatically enlarged the average grain size of the CuO/Cu_2_O nanocomposite as well (calculated by the Scherrer equation: *D* = 0.89*λ*/*β* cos *θ*).^28,29^ Hence, we concluded that the CuO/Cu_2_O heterojunction could be generated from a Cu sheet through the following two-step reactions:

1 Cu + O_2_ → Cu_2_O

2 Cu_2_O + O_2_ → CuO

The surface-contributed elements of CuO/Cu_2_O-3 were further investigated by X-ray photoelectron spectroscopy (XPS). As shown in Fig. 1C, the presence of peaks related to the C, Cu and O elements was clearly observed in the full scan spectra, and the relative contents of these elements are presented in Table S1;† moreover, the peak of C situated at 284.6 eV originated from the instrument.^30^ The binding energies of 932.4 and 952.4 eV are the split signals of Cu2p_3/2_ and Cu2p_1/2_, revealing that the Cu element is in the form of Cu^+^ in the composite, respectively (Fig. 1D); in addition to this, the neighbouring small peaks situated at 934.5 and 954.5 eV are assigned to Cu^2+^, which thereby shows a characteristic shake-up peak.^31^ The ratio of Cu^2+^/Cu^+^ was further investigated though quantitative analysis, as shown in Fig. 1D, and in the optimized CuO/Cu_2_O-3 composite, the ratio of CuO/Cu_2_O was found to be about 1 : 8.6, with the detailed data shown in Table S1.† The peaks at 530.4 and 531.8 eV are ascribed to the lattice oxygen and adsorbed oxygen, respectively (Fig. 1E).^32^ These results imply the successful construction of the CuO/Cu_2_O heterojunction, which are consistent with those of the XRD analysis.

A UV-Vis diffuse reflectance spectrum was obtained to evaluate the light absorption capacity of the CuO/Cu_2_O-3 composite. As shown in Fig. 1F, the strong absorption from 500 to 600 nm is attributed to the characteristic absorption of Cu_2_O. Furthermore, the spectrum of CuO/Cu_2_O-3 exhibits an absorption edge at 729 nm, which embodies the characteristic absorption of CuO with an indirect band gap, and the band gap of CuO has been estimated to be 1.7 eV according to the previously reported formula *E*_g_ = 1290/*λ*_max_.^33,34^ Therefore, the composite exhibits excellent light absorption across the visible spectrum. Moreover, it was found that different CuO/Cu_2_O composites showed the same light absorption onset for CuO (Fig. S1†); this revealed that CuO was successfully derived from Cu_2_O as a surface layer.

**Fig. 2:**
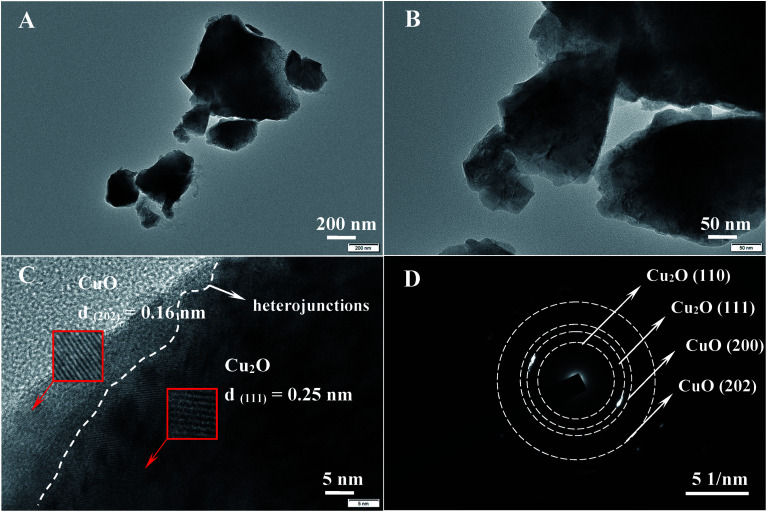
(A–C) TEM images and (D) SAED pattern of the CuO/Cu_2_O-3 sample.

The morphology of CuO/Cu_2_O-3 was evaluated using transmission electron microscopy (TEM), and the results are shown in Fig. 2. It can be observed that CuO/Cu_2_O-3 is composed of nanoparticles with a bulk structure (Fig. 2A and B). Importantly, Fig. 2C clearly confirms the presence of CuO/Cu_2_O heterojunctions in CuO/Cu_2_O-3, which further indicates that this product can act as a nanocomposite with a strong synergistic effect instead of a simple mixture. Moreover, the generated CuO can cover Cu_2_O as a protection layer, which is in good agreement with the results of the UV-Vis analysis. In addition, the lattices measured from the selected areas illustrate the spacings of 0.16 nm and 0.25 nm, corresponding to the (202) lattice facet of monoclinic CuO and the (111) lattice plane of cubic Cu_2_O.^35,36^ In addition to this, the selected area electron diffraction (SAED) pattern of CuO/Cu_2_O-3 was obtained, as shown in Fig. 2D. The diffraction rings from small to large separately signify Cu_2_O (110), Cu_2_O (111), CuO (200) and CuO (202), indicating a highly crystallized Cu_2_O and CuO structure.

### Photocatalytic applications of the CuO/Cu_2_O nanocomposites

**Fig. 3:**
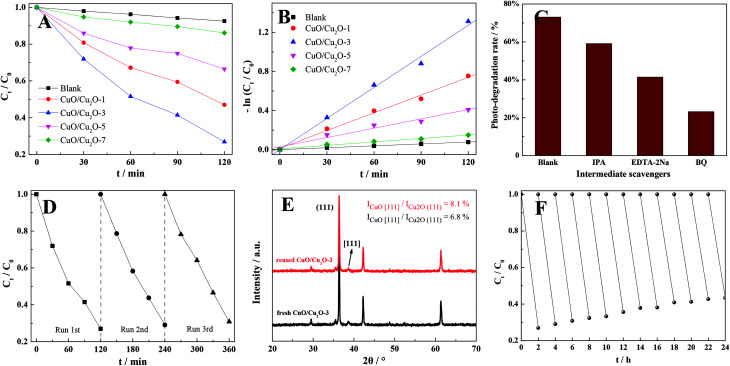
(A) Photocatalytic RhB removal profiles and (B) kinetics for different samples under visible light irradiation; (C) capture experiments conducted using isopropanol (IPA), ethylenediaminetetraacetic acid (EDTA-2Na), and benzoquinone (BQ) at 1.0 Mm, which were used as the hydroxyl radical (·OH) scavenger, hole (h^+^) scavenger, and superoxide radical (·O_2_^−^) scavenger to be injected into the reaction system, respectively; (D) stability test results of CuO/Cu_2_O-3 for three runs under the same condition; (E) XRD patterns of CuO/Cu_2_O-3 before and after photocatalytic removal; and (F) long-time stability test results of CuO/Cu_2_O-3 for 24 h.

To assess the photocatalytic performances of the different as-prepared samples, organic pollutant removal (RhB) experiments were conducted under visible-light irradiation. As shown in Fig. 3A, the RhB removal efficiencies were significantly influenced by the annealing time of the CuO/Cu_2_O nanocomposites, and highest RhB removal (*ca.* 73%) was obtained using the samples that were annealed for 3 h. In addition to this, the efficiency of dye removal over CuO/Cu_2_O-3 was further compared with those of the previously reported Cu-based photocatalysts. The developed composite could display a certain advantage for dye photodegradation (high concentration of RhB) as compared to those synthesized *via* conventional liquid-phase methods and even some other solid-phase methods (Table S2†). Moreover, based on the kinetic equation −ln(*C*_*t*_/*C*_0_) = *kt* + *b*,^37,38^ the pseudo-first-order reaction kinetics for the photocatalytic RhB removal over the CuO/Cu_2_O composites were calculated, and the results are shown in Fig. 3B. As indicated by the results, the photocatalytic removal rate constants were 0.65 × 10^−3^, 6.1 × 10^−3^, 10.6 × 10^−3^, 3.2 × 10^−3^ and 1.2 × 10^−3^ min^−1^ for the blank, CuO/Cu_2_O-1, CuO/Cu_2_O-3, CuO/Cu_2_O-5 and CuO/Cu_2_O-7 samples, respectively. In addition, the capture experiments were carried out to elucidate the nature and relative roles of different intermediates in the RhB photo-decomposition (Fig. 3C). Accordingly, the RhB removal reactions could be obviously restrained by BQ, manifesting that ·O_2_^−^ the main active substance of CuO/Cu2O-3 in the photocatalytic removal of the dye. Importantly, photocatalytic stability, being one of the requirements for large-scale application, was investigated using CuO/Cu_2_O-3, which subsequently exhibited only 4% deactivation of RhB removal after three runs when compared with the initial case; this manifested outstanding photocatalysis robustness of the proposed photocatalyst (Fig. 3D); furthermore, it was found that the crystal phase of CuO/Cu_2_O-3 had no distinct variation before and after photocatalysis, showing a favorable structural stability (Fig. 3E). Moreover, Fig. 3F displays the results of the longer photostability tests for CuO/Cu_2_O-3 to further demonstrate the potential of this photocatalyst for industrial applications. As observed, a gradual decrease in the rate of RhB removal was found due to the oxidation of Cu_2_O, slightly challenging the stability of the composite. Fortunately, it was observed that CuO/Cu_2_O-3 presented only 16.5% deactivation of RhB removal after twelve runs. The favorable stability might be attributed to the formation of CuO as a protective layer over Cu_2_O, effectively preventing the rapid oxidation of Cu_2_O.

**Fig. 4:**
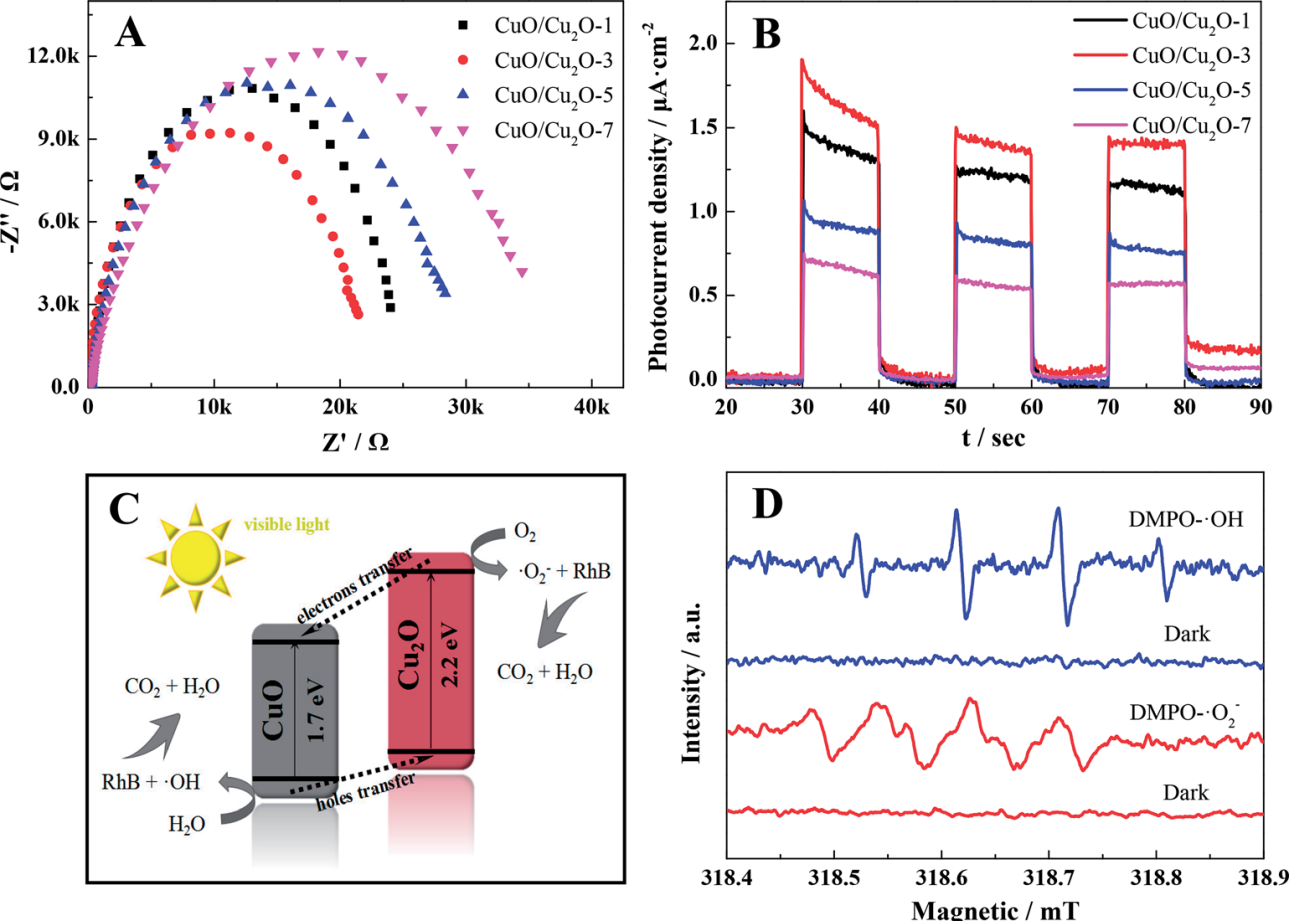
(A) Electrochemical impedance spectra and (B) transient photocurrent responses of different samples; (C) possible photocatalytic reaction mechanism of the CuO/Cu_2_O-3 composite; (D) DMPO spin-trapping ESR spectra of the CuO/Cu_2_O-3 composite in methanol and water for ·O_2_^−^ and ·OH before and after 10 min illumination, respectively.

The conspicuous enhancement in the photocatalytic activity is usually ascribed to the improved charge carrier behaviors. For this purpose, the transfer and separation capacities of the photogenerated carriers of different as-prepared samples were explored by electrochemical impedance spectroscopy and transient photocurrent responses, respectively.^39,40^ As shown in Fig. 4A, the radius of CuO/Cu_2_O-3 was much smaller than those of other CuO/Cu_2_O composites. This was attributed to the lower resistance and shorter transport distance in the case of CuO/Cu_2_O-3, indicating that the CuO/Cu_2_O-3 heterojunction had accelerated carrier migration at the interface between CuO and Cu_2_O. Moreover, it was apparently found in Fig. 4B that the CuO/Cu_2_O-3 composite exhibited an obviously boosted photocurrent density as compared to other CuO/Cu_2_O composites. This implied that the presence of moderate CuO was beneficial to the interfacial carrier behaviors and led to boosted carrier migration and separation of the CuO/Cu_2_O heterojunction for improved RhB removal. However, it was observed that excessive CuO might serve as recombination centers of charge carriers, hindering the migration and separation of electron–hole pairs. Furthermore, the charge carrier behaviors could be affected by the grain sizes of the CuO/Cu_2_O composites. With an increase in the annealing time, the average grain size of the CuO/Cu_2_O composite was dramatically enlarged, facilitating the recombination of electron–hole pairs. Hence, we could infer that the optimum condition for the thermal exfoliation of the Cu sheet was the annealing time of 3 h.

Moreover, a possible photocatalytic mechanism of the CuO/Cu_2_O composite was proposed, as schematically illustrated in Fig. 4C, according to the data presented in Table S3.† Accordingly, the reported conduction band (CB) potential of Cu_2_O (−0.28 eV) is more negative than that of CuO (0.46 eV), whereas the valence band (VB) potential of CuO (2.16 eV) is more positive than that of Cu_2_O (1.92 eV); this demonstrates that a desired heterojunction between CuO and Cu_2_O can be established due to this well-matched band structure. Hence, when the CuO/Cu_2_O composite is exposed to visible light, the excited photogenerated electrons transfer from the CB of Cu_2_O to that of CuO and the holes simultaneously transfer from the VB of CuO to that of Cu_2_O; this leads to an improved separation and transfer of photogenerated charge carriers. More importantly, CuO and Cu_2_O thereby serve as different active sites for electron reduction and hole oxidation, which can restore O_2_ into superoxide radicals (·O_2_^−^) and oxidize H_2_O into hydroxyl radicals (·OH), respectively. Subsequently, these macromolecular organic pollutants can be degraded by ·O_2_^−^ and ·OH into micromolecular organics and ultimately be mineralized into CO_2_ and H_2_O due to the strong oxidizing capacity of ·O_2_^−^ and ·OH, respectively.

In addition, the electron spin resonance (ESR) technique was employed to confirm the existence of ·O_2_^−^ and ·OH in the photocatalytic reactions,^41,42^ and the results are shown in Fig. 4D. No signals were observed in the dark. However, the strong characteristic peaks of DMPO–·O_2_^−^ and DMPO–·OH were separately found in methanol and water when the CuO/Cu_2_O composites were illuminated by visible light, suggesting that both ·O_2_^−^ and ·OH were continually generated during the photocatalytic organic pollutant removal.

### Cycled thermal exfoliation of the Cu sheets

**Fig. 5:**
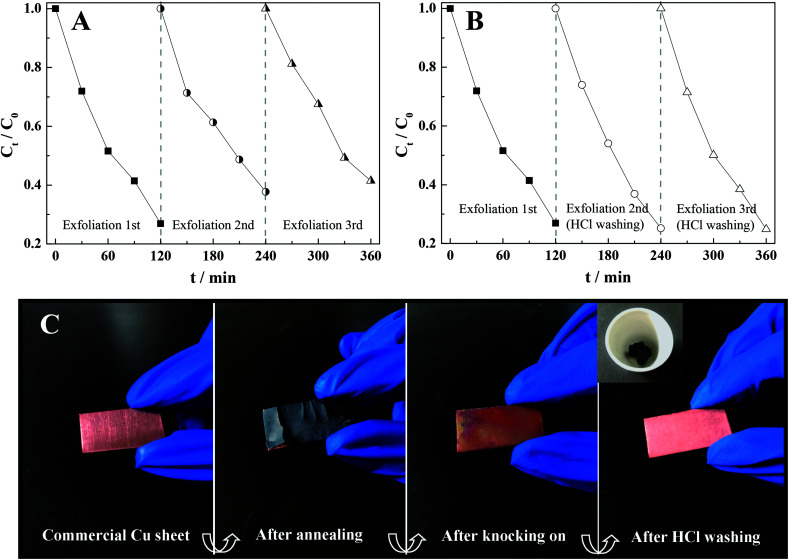
(A) Stability test results of CuO/Cu_2_O-3 for cycled thermal exfoliation under visible light irradiation; (B) stability test results of CuO/Cu_2_O-3 for cycled thermal exfoliation after HCl washing under the same conditions; and (C) schematic of the fabrication procedure towards the CuO/Cu_2_O nanocomposite powders.

Reproducibility of thermal exfoliation can be another critical assessment for promising large-scale applications. Consequently, we repeatedly exfoliated the appointed Cu sheet three times at 500 °C during 3 h to obtain different CuO/Cu_2_O-3 composites, which were subsequently employed for photocatalysis. As shown in Fig. 5A, a slightly decreased photocatalytic activity of the CuO/Cu_2_O-3 composite was observed after several thermal exfoliations, which still demonstrated preferable photocatalytic stability. Moreover, the results of XRD indicated that the content of CuO in CuO/Cu_2_O-3 was increased when compared with the initial content after exfoliating the Cu sheet several times (Fig. S2†). Accordingly, the deactivation of the CuO/Cu_2_O-3 composites might be attributed to incomplete exfoliation, resulting in the coating of copper oxide on the surface of the Cu sheet and thus hindering the subsequent exfoliation. To validate our conjecture, photocatalysis experiments were conducted using the CuO/Cu_2_O-3 composites with different exfoliated frequencies, which were washed with an HCl solution (1 mol L^−1^) every time before conducting the photocatalytic reactions. Amazingly, we found that the photocatalytic activities of CuO/Cu_2_O-3 were slightly enhanced with the increasing exfoliation times (Fig. 5B). Hence, we inferred that a thinner Cu sheet would lead to significantly high thermal exfoliation efficiency.

In addition, considering commercialization, the Cu sheet has a small volume and regular structure, which is convenient for storage and transportation. Especially, the CuO/Cu_2_O heterogeneous photocatalyst can be simply acquired in the powder form just by the annealing of the Cu sheets and the subsequent mechanical knock-on, and the synthetic evolution is shown in Fig. 5C. Note that this solid-phase reaction strategy seems more desirable due to low-cost raw materials, user-friendly operation and simple device structure, which may have feasible large-scale applications.

## Conclusions

In summary, we developed a facile thermal exfoliation strategy to fabricate CuO/Cu_2_O heterojunction powders using a commercial Cu sheet as a precursor. As expected, the contents of CuO in the nanocomposites could be increased by increasing the annealing time. The resultant CuO/Cu_2_O composite featured an outstanding visible light response and a characteristic absorption edge at 729 nm. Moreover, the optimized CuO/Cu_2_O nanocomposite demonstrated favorable photocatalytic activity and stability for organic pollutant (RhB) removal. More importantly, it has been found that CuO and Cu_2_O have well-matched band structures that are favourable for accelerated transfer and separation of charge carriers; moreover, CuO and Cu_2_O serve as different active sites for electron reduction and hole oxidation during photocatalysis. Furthermore, the presence of the ·O_2_^−^ and ·OH species with strong oxidability over nanocomposites was directly confirmed by the ESR analysis, revealing the reason for the significant photocatalytic removal of organic pollutants. In addition, the cycled thermal exfoliation experiments confirmed the excellent stability of the nanocomposites after multiple exfoliations from the Cu sheet, attesting a promising industrial value for photocatalytic practical applications. Moreover, this novel CuO/Cu_2_O nanocomposite might precede commercial TiO_2_ for large-scale applications due to its advantages such as low-cost raw materials, user-friendly operation, simple device structure, broad visible-light responses and strong active species production. Unlike the conventional liquid-phase synthesis, this study provided a cost-effective and convenient synthetic strategy for a heterogeneous photocatalyst, ultimately guiding the brand new perspectives for photocatalytic research.

## Experimental

### Preparation of the CuO/Cu_2_O heterogeneous photocatalyst

The CuO/Cu_2_O composites were prepared by the thermal exfoliation of commercial copper sheets. Commercial copper sheets (Cu, 99.5%) were obtained from Sinopharm Chemical Reagent Co. Ltd. (Shanghai, China). Typically, Cu sheets (cut into 3.0 × 1.5 cm^2^ pieces) were repeatedly rinsed with HCl solution (1 mol L^−1^) and ethanol to remove the surface oxide layer and then annealed at 500 °C at the heating rate of 5 °C min^−1^. Subsequently, the obtained CuO/Cu_2_O/Cu heterojunction sheets were immediately fetched from the muffle furnace. After naturally cooling the sheets down to room temperature, the CuO/Cu_2_O composites were exfoliated from the CuO/Cu_2_O/Cu sheets through a simple mechanical knock-on process and then ground into powders in an agate mortar. Based on the different annealing times (1, 3, 5 and 7 hours), the final products were denoted as CuO/Cu_2_O-1, CuO/Cu_2_O-3, CuO/Cu_2_O-5, and CuO/Cu_2_O-7, respectively.

### Characterization of photocatalytic materials

X-ray diffraction (XRD) patterns of the products were obtained by a diffractometer (Bruker-AXS/D8) using Kα radiation in the range of 20–70°. X-ray photoelectron spectra (XPS) were obtained by a spectrometer (ESCALAB/250Xi) using Al Kα radiation. The microstructures of the products were explored by transmission electron microscopy (TEM, JEOL/JEM-2100PLUS). The UV-Vis diffuse reflection spectra (DRS) of the products were measured by a spectrophotometer (Purkinje/TU-1901) fitted with an integrating sphere using BaSO_4_ as a reference. Electron spin resonance (ESR) measurements of the products were conducted by a spectrometer (JEOL/JES-FA200) in the presence of DMPO.

### Electrochemical and photocatalytic tests

Electrochemical impedance spectroscopy and transient photocurrent responses of different products were achieved by an electrochemical workstation (Shanghai Chenhua Technology Co./CHI 760D) using a Pt wire and Ag/AgCl electrode as the counter electrode and reference electrode, respectively. The working electrodes were made by dip-coating 10 μL of product slurry (5 mg mL^−1^) onto titanium plates (1 × 1 cm^2^), followed by air drying at 100 °C. The electrolyte was a KCl aqueous solution at a concentration of 0.1 M.

The organic pollutant removal (RhB) experiments were carried out using a photocatalytic online analysis system (Beijing PerfectLight Technology Co./LabSolar-IIIAG), and a 300 W Xe-lamp with a 420 nm cut-off (PLS-SXE300/300UV) was chosen as the visible light source as previously reported by our group.^43,44^ Briefly, 0.05 g powders were dispersed in 50 mL aqueous RhB (20 mg L^−1^) under sonication. Before illumination, the suspension was stirred for 30 min in the dark to reach an adsorption–desorption equilibrium. A 3 mL reaction solution was taken from the reactor at a certain time interval, and then, the photocatalyst was removed through centrifugation. The concentrations of RhB were determined by a UV-Vis spectrophotometer (TU-1901) at 554 nm.

## Conflicts of interest

There are no conflicts to declare.

## Supplementary Material

RA-009-C9RA06837F-s001

## References

[cit1] Chu C., Huang D., Zhu Q., Stavitski E., Spies J. A., Pan Z., Mao J., Xin H. L., Schmuttenmaer C. A., Hu S., Kim J. H. (2018). ACS Catal..

[cit2] Yan W., Liu X., Hou S., Wang X. (2019). Catal. Sci. Technol..

[cit3] Mishra P., Patnaika S., Parida K. (2019). Catal. Sci. Technol..

[cit4] Sui Y., Liu Q., Jiang T., Guo Y. (2017). Appl. Surf. Sci..

[cit5] Shen H., Ni D., Niu P., Zhou Y., Zhai T., Ma Y. (2017). Int. J. Hydrogen Energy.

[cit6] Zhou L., Zhang W., Chen L., Deng H., Wan J. (2017). Catal. Commun..

[cit7] Gao W., Zhang X., Su X., Wang F., Liu Z., Liu B., Zhan J., Liu H., Sang Y. (2018). Chem. Eng. J..

[cit8] Guo H., Niu C. G., Zhang L., Wen X. J., Liang C., Zhang X. G., Guan D. L., Tang N., Zeng G. M. (2018). ACS Sustainable Chem. Eng..

[cit9] Wang F., Wang Y., Feng Y., Zeng Y., Xie Z., Zhang Q., Su Y., Chen P., Liu Y., Yao K., Lv W., Liu G. (2018). Appl. Catal., B.

[cit10] Wang S., Guan B. Y., Lou X. W. D. (2018). J. Am. Chem. Soc..

[cit11] Liu J. Y., Fang W. J., Wei Z. D., Qin Z., Jiang Z., Shangguan W. F. (2018). Catal. Sci. Technol..

[cit12] Li Z., Zhou Z., Ma J., Li Y., Peng W., Zhang G., Zhang F., Fan X. (2018). Appl. Catal., B.

[cit13] Duan J., Zhao H., Zhang Z., Wang W. (2018). Ceram. Int..

[cit14] Singh G., Kumar M., Bhalla V. (2018). Green Chem..

[cit15] Li W., Feng X., Zhang Z., Jin X., Liu D., Zhang Y. (2018). Adv. Funct. Mater..

[cit16] Gong H., Zhang Y., Cao Y., Luo M., Feng Z., Yang W., Liu K., Cao H., Yan H. (2018). Appl. Catal., B.

[cit17] Li D., Zuo S., Xu H., Zan J., Sun L., Han D., Liao W., Zhang B., Xia D. J. (2018). J. Colloid Interface Sci..

[cit18] Niu W., Shi J., Ju L., Li Z., Orlovskaya N., Liu Y., Yang Y. (2018). ACS Catal..

[cit19] Zhuang T. T., Pang Y., Liang Z. Q., Wang Z., Li Y., Tan C. S., Li J., Dinh C. T., Luna P. D., Hsieh P. L., Burdyny T., Li H. H., Liu M., Wang Y., Li F., Proppe A., Johnston A., Nam D. H., Wu Z. Y., Zheng Y. R., Ip A. H., Tan H., Chen L. J., Yu S. H., Kelley S. O., Sinton D., Sargent E. H. (2018). Nat. Catal..

[cit20] Lee J. E., Kim D. Y., Lee H. K., Park H. J., Ma A., Choi S. Y., Lee D. S. (2019). Sens. Actuators, B.

[cit21] Sandroni M., Gueret R., Wegner K. D., Reiss P., Fortage J., Aldakov D., Collomb M. N. (2018). Energy Environ. Sci..

[cit22] Xie T., Zhang Y., Yao W., Liu Y., Wang H., Wu Z. (2019). Catal. Sci. Technol..

[cit23] Chen B., Meng Y., Sha J., Zhong C., Hu W., Zhao N. (2017). Nanoscale.

[cit24] Jiang D., Xue J., Wu L., Zhou W., Zhang Y., Li X. (2017). Appl. Catal., B.

[cit25] Li H., Su Z., Hu S., Yan Y. (2017). Appl. Catal., B.

[cit26] Tan C. S., Hsu S. C., Ke W. H., Chen L. J., Huang M. H. (2015). Nano Lett..

[cit27] Pradhan A. C., Uyar T. (2017). ACS Appl. Mater. Interfaces.

[cit28] Gomathisankar P., Hachisuka K., Katsumata H., Suzuki T., Funasaka K., Kaneco S. (2013). ACS Sustainable Chem. Eng..

[cit29] Chen X., Li L., Zhang W., Li Y., Song Q., Zhang J., Liu D. J. (2016). J. Mol. Catal. A: Chem..

[cit30] Wei Z., Benlin D., Fengxia Z., Xinyue T., Jiming X., Lili Z., Shiyin L., Leung D. Y. C., Sun C. (2018). Appl. Catal., B.

[cit31] Dubale A. A., Pan C. J., H Tamirat A. G., Chen M., Su W. N., Chen C. H., Rick J., Ayele D. W., Aragaw B. A., Lee J. F., Yang Y. W., Hwang B. J. (2015). J. Mater. Chem. A.

[cit32] Mishra A. K., Pradhan D. (2016). Cryst. Growth Des..

[cit33] Shenawi-Khalil S., Uvarov V., Fronton S., Popov I., Sasson Y. (2012). J. Phys. Chem. C.

[cit34] Chen X., Zhang W., Zhang L., Feng L., Wen J., Yang J., Zhang C., Jiang J., Wang H. (2019). Appl. Surf. Sci..

[cit35] Verma A., Jaihindh D. P., Fu Y. P. (2019). Dalton Trans..

[cit36] Liu J., Ke J., Li D., Sun H., Liang P., Duan X., Tian W., Tade M. O., Liu S., Wang S. (2017). ACS Appl. Mater. Interfaces.

[cit37] Yang Z. M., Huang G. F., Huang W. Q., Wei J. M., Yan X. G., Liu Y. Y., Jiao C., Wan Z., Pan J. (2014). J. Mater. Chem. A.

[cit38] Jiang J., Zhang X., Sun P., Zhang L. (2011). J. Phys. Chem. C.

[cit39] Xiao Y., Tian G., Li W., Xie Y., Jiang B., Tian C., Zhao D., Fu H. (2019). J. Am. Chem. Soc..

[cit40] Kim C., Cho K. M., Al-Saggaf A., Gereige I., Jung H. T. (2018). ACS Catal..

[cit41] Jiang Z., Wan W., Li H., Yuan S., Zhao H., Wong P. K. (2018). Adv. Mater..

[cit42] Wang Q., Zhou H., Liu X., Li T., Jiang C., Song W., Chen W. (2018). Environ. Sci.: Nano.

[cit43] Chen X., Li L., Zhang W., Li Y., Song Q., Dong L. (2016). ACS Sustainable Chem. Eng..

[cit44] Zhang J., Li L., Xiao Z., Liu D., Wang S., Zhang J., Hao Y., Zhang W. (2016). ACS Sustainable Chem. Eng..

