# Supplementary material for: Facile thermal exfoliation of Cu sheets towards the CuO/Cu2O heterojunction: a cost-effective photocatalyst with visible-light response for promising sustainable applications
Source: RSC Adv. 2019 Oct 17;9(57):33395–402. doi: 10.1039/c9ra06837f (PMC9073361; doi:10.1039/c9ra06837f)
Supplement: RA-009-C9RA06837F-s001 [file RA-009-C9RA06837F-s001.pdf]

Electronic Supplementary Material (ESI) for RSC Advances.
This journal is © The Royal Society of Chemistry 2019

Electronic Supplementary Information:

Facile Thermal Exfoliation of Cu Sheets towards CuO/Cu_2O Heterojunction: A Cost-effective Photocatalyst with Visible-light Response for Promising Sustainable Applications

Yixuan Li [a], Xi Chen [a, b], Li Li [a, b, c *]

[a] College of Chemistry and Chemical Engineering, Qiqihar University, Qiqihar, Heilongjiang 161006, P. R. China.

[b] College of Chemistry and Chemical Engineering, Harbin Institute of Technology, Harbin, Heilongjiang 150090, P. R. China.

[c] College of Materials Science and Engineering, Qiqihar University, Qiqihar, Heilongjiang 161006, P. R. China.

[*] Corresponding author: Li Li, female, Ph.D., Professor, doctoral tutor, mainly engaged in the preparation and photocatalytic properties of nanocomposites, +86 0452-2738351. E-mail: qqhrll@163.com; qqhrlili@126.com (L.Li).

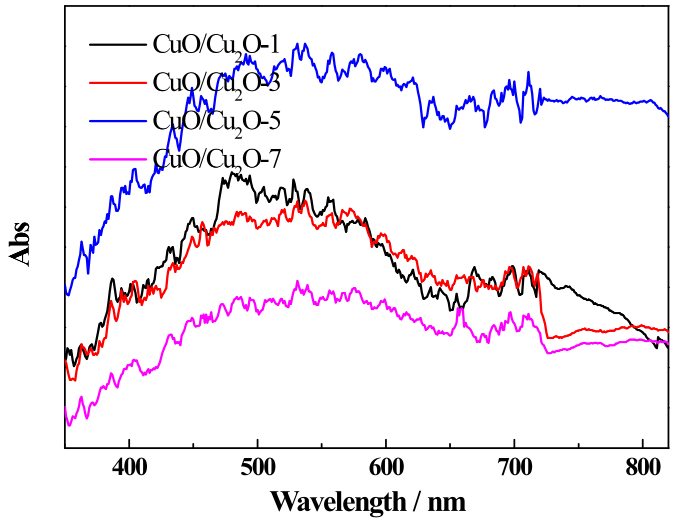

Fig. S1 UV−Vis DRS spectrum of different CuO/Cu_2O composites.

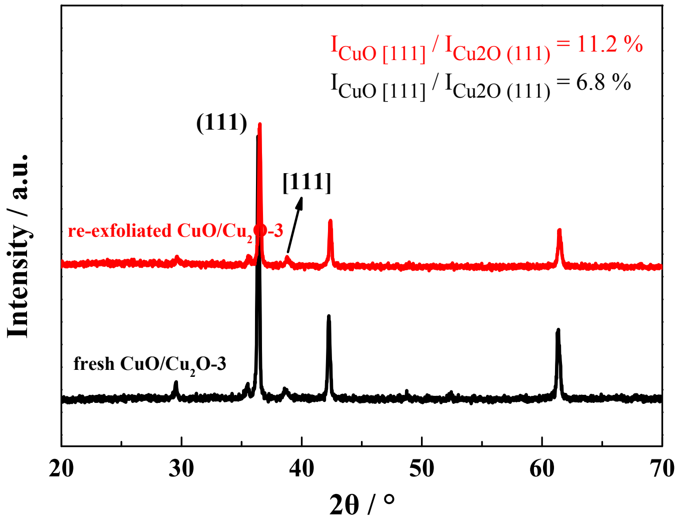

Fig. S2 XRD patterns of CuO/Cu_2O-3 before and after photocatalytic removal for cycled thermal exfoliation.

Tab. S1 Element content analysis of CuO/Cu_2O-3 composite from XPS spectra (Atomic %)

Sample	C/%	O/%	Cu^{+}/%	Cu^{2+}/%
CuO/Cu_2O-3	61.99	28.77	8.28	0.96

Tab. S2 Comparison of different performances of Cu-based photocatalysts for dye degradation

Catalyst	synthetic method	Light source	Catalyst loading (g)	Dye	Concentration (mg/L)	Time (min)	Efficiency
CuO	Solid phase[1]	UV lamp	0.5	MB	10	210	33%
Cu/CuO	Liquid phase[2]	UV lamp	0.02	MB	10	50	15%
Cu/Cu_2O	Solid phase[3]	Xe lamp (420 nm cut-off)	0.28	MB	20	120	67%
$Cu@Cu_2O$	Liquid phase[4]	UV lamp	0.01	MB	10	50	4.7%
CuO/Cu_2O	Gas phase[5]	Xe lamp (420 nm cut-off)	Not detectable	MB	10	240	90%
CuO/Cu_2O	Liquid phase[6]	Xe lamp (420 nm cut-off)	0.015	MO	6.5	120	95%
$CuO/Cu_2O/Cu$	Liquid phase[7]	Blue LED	0.015	MB	10	90	92%
**CuO/Cu_2O (This work)**	**Solid phase**	**Xe lamp (420 nm cut-off)**	**0.05**	**RhB**	**20**	**120**	**73%**

Tab. S3 The energy band gaps (Eg), conduction band (E_{CB}) and valence band potentials (E_{VB}) of CuO and Cu_2O

Sample	Eg/eV	E_{CB}/eV	E_{VB}/eV
CuO	1.7	0.46	2.16
Cu_2O	2.2	-0.28	1.92

*These values were obtained from the previous representative reports.[8,9]

References

(1) R. Saravanan, S. Joicy, V. Gupta, V. Narayanan, A. Stephen, Mater. Sci. Eng. C 2013, **33,** 4725-4731.

(2) L. Xu, C. Srinivasakannan, J. Peng, L. Zhang, D. Zhang, J. Alloy Compd. 2017, **695,** 263-269.

(3) W. Chen, Z. Fan, Z. Lai, J. Mater. Chem. A 2013, **1,** 13862-13868.

(4) L. Xu, C. Srinivasakannan, J. Peng, M. Yan, D. Zhang, L. Zhang, Appl. Surf. Sci. 2015, **331,** 449-454

(5) N.D. Khiavi,, R. Katal,, S.K. Eshkalak, S.M. Panah, S. Ramakrishna, H.J. Yong, Nanomaterials 2019, **9,** 1011.

(6) D. Jiang, J. Xue, L. Wu, W. Zhou, Y. Zhang, X. Li, Appl. Catal. B Environ. 2017, **211,** 199-204.

(7) S. Mosleh, M.R. Rahimi, M. Ghaedi, K. Dashtian, S. Hajati, Ultrasomn. Sonochem. 2018, **40,** 601-610.

(8) H. Li, Z. Su, S. Hu, Y. Yan, Appl. Catal. B Environ. 2017, **207,** 134-142.

(9) Y. Xu, M.A.A. Schoonen, Am. Mineral 2000, **85,** 543-556.
